# Supplementary material for: The influence of physical exercise on emotional management ability in college students: A serial mediation model of exercise adherence and psychological resilience
Source: PLoS One. 2025 Nov 10;20(11):e0334288. doi: 10.1371/journal.pone.0334288 (PMC12599973; doi:10.1371/journal.pone.0334288)
Supplement: Attachment — Submitted filename: Response to Editor and Reviewers.docx [file pone.0334288.s002.docx]

Manuscript Number: **PONE-D-25-33803**

Title: **The Influence of Physical Exercise on Emotional Management Ability in College Students: A Serial Mediation Model of Exercise Adherence and Psychological Resilience**

Journal: **PLOS One**

**Point-by-point Responses to Editor**

Dear Editor and dear reviewers,

Thank you very much for your comments and professional advice. These opinions help to improve academic rigor of our manuscript. Based on your suggestion and request, we have made corrected modifications on the revised manuscript. Here are point-by-point responses to your comments, We hope that our work can be improved again.

Sincerely,

On behalf of all authors

**Comment 1:**

Please ensure that your manuscript meets PLOS ONE's style requirements, including those for file naming. The PLOS ONE style templates can be found at

https://journals.plos.org/plosone/s/file?id=wjVg/PLOSOne_formatting_sample_main_body.pdf and

https://journals.plos.org/plosone/s/file?id=ba62/PLOSOne_formatting_sample_title_authors_affiliations.pdf

**Response 1:**

**Thank you very much for your detailed review and valuable comments. I have reformatted the manuscript according to PLOS ONE’s style templates, including file naming conventions.**

**Comment 2:**

We note that the grant information you provided in the ‘Funding Information’ and ‘Financial Disclosure’ sections do not match.

When you resubmit, please ensure that you provide the correct grant numbers for the awards you received for your study in the ‘Funding Information’ section.

**Response 2:**

**Thank you very much for your insightful comments and valuable recommendations concerning our manuscript. The funder (2024 Jiangsu Philosophy and Social Science Research Project, No. 2024SJYB1253) only provide financial support for research and distribution of questionnaires, and are not involved in any research activities. The revised funder statement has been included in the cover letter and manuscript.**

**Comment 3:**

In the online submission form you indicate that your data is not available for proprietary reasons and have provided a contact point for accessing this data. Please note that your current contact point is a co-author on this manuscript. According to our Data Policy, the contact point must not be an author on the manuscript and must be an institutional contact, ideally not an individual. Please revise your data statement to a non-author institutional point of contact, such as a data access or ethics committee, and send this to us via return email. Please also include contact information for the third party organization, and please include the full citation of where the data can be found.

**Response 3:**

**Thank you very much for your close attention to the details of our manuscript. We have revised the data statement to indicate that ethical approval was granted by the Ethics Review Committee of Nantong University, and that the data are available upon request from kjc@ntu.edu.cn. Thank you again for your valuable feedback.**

**Comment 4:**

Your ethics statement should only appear in the Methods section of your manuscript. If your ethics statement is written in any section besides the Methods, please move it to the Methods section and delete it from any other section. Please ensure that your ethics statement is included in your manuscript, as the ethics statement entered into the online submission form will not be published alongside your manuscript.

**Response 4:**

**Thank you for highlighting the placement of the ethics statement. We have moved it entirely to the Methods section (lines 266–270) and removed all duplicate text from the Introduction, Results, and any other sections, so that only one complete statement remains. This revision includes the approval number, the approving body (Ethics Review Committee of Nantong University), and the informed-consent procedure, and meets the journal’s formatting requirements. Thank you again for your careful review and guidance.**

**Comment 5:**

Please include captions for your Supporting Information files at the end of your manuscript, and update any in-text citations to match accordingly. Please see our Supporting Information guidelines for more information: http://journals.plos.org/plosone/s/supporting-information.

**Response 5:**

**Thank you very much for your insightful comments and valuable recommendations concerning our manuscript.** **I have added captions for figures and tables as required support information section.**

**Comment 6:**

If the reviewer comments include a recommendation to cite specific previously published works, please review and evaluate these publications to determine whether they are relevant and should be cited. There is no requirement to cite these works unless the editor has indicated otherwise.

**Response 6:**

**Thank you very much for your insightful comments and valuable recommendations concerning our manuscript. References have been verified. No retracted papers were cited.** **Also, the references cited in this paper are all from the last three to five years with some classic literature.**

**Point-by-point Responses to Reviewer 1**

**Comment 1:**

Data Reporting: While the manuscript specifies the number of valid responses, it should also report the total number of surveys distributed/collected and the valid response rate (i.e., valid responses as a percentage of total collected). This is essential for assessing potential selection bias.

**Response 1:**

**Thank you very much for pointing out this issue, which is extremely important for enhancing the quality and applicability of our research. For more details, please refer to lines 252-266 of the manuscript.**

**Comment 2:**

Measurement Norms: It is unclear whether the instruments measuring "Emotional Management" and "Exercise Adherence" have established normative data specifically for the Chinese college student population. The validity of subsequent statistical analyses hinges on confirming whether such population-specific norms exist and were used.

**Response 2:**

**Thank you very much for your question, which is of great help to our manuscript. The measurement tools for “emotional management” and “exercise adherence” used in this study have been validated among Chinese college students and have good reliability and validity. The statistical analysis based on the data collected by these tools is valid. We also conducted a pretest and localization adjustments in our study to further ensure the applicability of the tools and the reliability of the analysis results. For details, please refer to line 307-311 and line 324-329 of the manuscript.**

**Comment 3:**

Statistical Analysis: The use of Pearson correlation assumes interval-level data. If the "Physical Exercise" variable (categorized as low, medium, high volume) is treated as ordinal, alternative correlation analyses appropriate for ordinal data (e.g., Spearman's rho or Kendall's tau) should be employed. The authors must explicitly address this methodological choice.

**Response 3:**

**Thank you very much for raising this point. In our study, physical exercise was quantified using a composite score, which is why we opted for Pearson. Of course, the approach you suggested is highly valuable, and we will make greater use of it in our future work.** **Thank you again for your careful review and guidance.**

**Comment 4:**

Sample Representation: The representation of fourth-year students (1.3%) is statistically insufficient for meaningful analysis. I strongly recommend combining third- and fourth-year students into a single "Upperclassmen" category to contrast with "Underclassmen" (years 1-2), ensuring adequate group size for comparisons.

**Response 4:**

**Thank you for highlighting the low representation of fourth-year students (1.3%). Following your recommendation, we combined third- and fourth-year participants into a single "senior" group and contrasted it with the "junior" group (first- and second-years). After pooling, both sub-samples exceed 5% of the total, meeting the minimum requirement for subsequent statistical tests and markedly improving the precision of effect-size estimates. All related tables, figures, and text have been updated; please see lines 372-401 and revised Tables 1-3 for details. Thank you again for your careful review and guidance.**

**Comment 5:**

Data Accuracy (Table 3): The reported value η² = 0 is highly improbable. Effect sizes (eta-squared) approaching zero are rare in practice; this warrants careful recalculation and verification by the authors.

**Response 5:**

**Thank you for highlighting the anomaly of η² = 0 in Table 3. We immediately re-examined the raw data and SPSS output and found that the value was due to the software’s default two-decimal rounding. In the revised manuscript we now report all corresponding effect sizes as “< 0.001” and have standardised the decimal places throughout. Additionally, we double-checked every other η², F value, and degree of freedom in Table 3 to ensure complete accuracy. The updated results can be found in lines 372–374 and in the revised Table 2. Thank you again for your meticulous review and helpful feedback.**

**Point-by-point Responses to Reviewer 2**

**Comment 1:**

Address the Negative Direct Effect: The manuscript reports a negative direct effect of PE on EMA in the regression analysis, which contradicts the positive correlation found in the correlation analysis. Clarify this discrepancy by:

- Discussing potential suppressor variables or confounding factors that might explain this effect.

- Conducting additional analyses to explore potential interactions between PE, EA, PR, and other relevant variables.

**Response 1:**

**1. Thank you for raising the important question regarding the contradiction between the positive correlation and the negative direct effect of physical exercise (PE) on emotional management ability (EMA) in our study. When examining the relationship between PE volume and EMA, the study revealed a significant negative predictive relationship (β = -0.009), failing to support research hypothesis H1. However, correlation analysis indicated a significant positive correlation between these variables (r = 0.164). This seemingly contradictory finding may arise from the combined effects of multiple factors. The negative prediction coefficient in the regression analysis may suggest the presence of confounding variables. Within the multiple regression model, PE volume may exhibit collinearity with other variables (e.g., stress levels and sleep quality). These variables might be prioritized in the model, thereby attenuating the direct influence of PE volume on EMA. Conversely, the positive correlation in the correlation analysis may reflect a direct association between PE volume and EMA, suggesting that PE can enhance EMA. When controlling for additional variables, other, more prominent factors might obscure or reverse this direct relationship. Sample characteristics, the sensitivity of measurement tools, and limitations inherent in the statistical methods employed may also contribute to this inconsistency. Therefore, when interpreting this phenomenon, carefully considering model complexity, variable interactions, and potential biases within the study design is warranted to elucidate the complex relationship between PE volume and EMA fully. For details, please refer to line 432-448 of the manuscript.**

**2. We appreciate your suggestion to include diagnostics for multicollinearity and omitted variable bias, and we have conducted these analyses to provide a clearer explanation.**

**To address the potential issue of multicollinearity, we calculated the Variance Inflation Factors (VIF) for all predictors in our regression model. The VIF values for PE, EMA, exercise adherence (EA), and psychological resilience (PR) were all found to be less than 5, indicating that multicollinearity is not a significant concern in our model. Specifically, the VIF values are as follows: PE (1.402), EA (1.359), PR (1.132), gender (1.174), and grade (1.008). These results suggest that the high intercorrelations among variables are not inflating the standard errors to a problematic extent.**

**We also conducted a stepwise regression analysis to further investigate the role of the mediating variables (EA and PR) in the relationship between PE and EMA. The results indicate that the contradiction may arise from the substantial mediating effects of EA and PR. In our model, PE has a significant positive effect on EA (β = 0.202, p < 0.001) and PR (β = 0.010, p < 0.001). EA, in turn, has a significant positive effect on PR (β = 0.149, p < 0.001) and EMA (β = 0.206, p < 0.001). PR also has a significant positive effect on EMA (β = 0.287, p < 0.001). When these mediating effects are taken into account, the direct effect of PE on EMA becomes negative (β = -0.009, p < 0.001).**

**In summary, the contradiction between the positive correlation and the negative direct effect of PE on EMA is likely due to the substantial mediating effects of EA and PR. These mediating variables carry a large amount of effect, overshadowing the true direct effect of PE on EMA. We have included the VIF values to confirm that multicollinearity is not a significant issue in our model. We hope this detailed explanation clarifies the issue and addresses your concerns.** **The specific revisions can be found on lines 390-402 and Table 6.**

**Comment 2:**

Strengthen the Discussion Section:

- Elaborate on the practical implications of the findings for college students and higher education institutions.

- Provide specific recommendations for interventions to promote physical exercise, enhance exercise adherence, and improve psychological resilience among college students.

- Address the limitations of the study more thoroughly and suggest avenues for future research.

**Response 2:**

**Thank you for your systematic suggestions regarding practical implications, intervention recommendations, and limitations/future directions. We have expanded the discussion to clarify the findings’ practical significance for university students and higher-education institutions; please see lines 469–485 for details. In addition, we have added a limitation note suggesting that future studies further explore potential confounding variables; see lines 607–612.**

**Comment 3:**

Provide More Detail on Measures:

- Include more information about the validity and reliability of the scales used in the study.

**Response 3:**

**Thank you for your question. In response, we have provided more detailed supplementary information regarding the resolution of this issue. For specifics, please refer to lines 307 to 311 and 324 to 329 of the article.**

**Comment 4:**

Consider Alternative Analytical Approaches:

- Explore the use of structural equation modeling (SEM) to examine the relationships between PE, EA, PR, and EMA more comprehensively.

**Response 4:**

**We sincerely appreciate your interest in our work and your insightful recommendation to employ structural equation modeling (SEM) so as to examine the associations among PE, EA, PR and EMA in a more integrative fashion. We fully concur that clarifying how these four constructs interrelate is essential for a comprehensive representation of our theoretical framework and empirical findings.**

**Nevertheless, several pragmatic obstacles prevented us from implementing SEM in the present study. The specific characteristics of our dataset—namely, pronounced non-normality, high multicollinearity among latent indicators, and a limited sample-to-parameter ratio—rendered conventional SEM estimators (e.g., maximum likelihood, weighted least squares) unstable and prone to inadmissible solutions. After extensive robustness checks (including item parceling, asymptotically distribution-free estimators, and Bayesian SEM with informative priors), goodness-of-fit indices remained below acceptable thresholds (CFI < 0.85, RMSEA > 0.10), and residual diagnostics revealed persistent specification error. Consequently, we concluded that SEM would not yield valid or replicable parameter estimates under the current data conditions.**

**We therefore adopted a more conservative analytical strategy, reporting zero-order correlations and hierarchical regression models while explicitly acknowledging their inability to model reciprocal influences or latent measurement error. Although this approach is less elegant than SEM, we believe it provides the most transparent and defensible portrayal of the PE–EA–PR–EMA associations given the present constraints. Recognizing the theoretical value of SEM, we are now collecting a larger, multilevel longitudinal sample that will satisfy minimum requirements for asymptotic robustness and allow us to test the full mediation–moderation network you propose in subsequent work.**

**Comment 5:**

Ensure Clarity and Consistency:

- Review the manuscript carefully to ensure clarity and consistency in terminology, formatting, and reporting of statistical results.

**Response 5:**

**We sincerely thank you for your meticulous review and invaluable comments on our manuscript. We have carefully examined the paper to ensure clarity and consistency in terminology, formatting, and the reporting of statistical results.**

**Comment 7:**

Theoretical Framework: While the study mentions social cognitive theory, expanding on this or integrating other relevant theories (e.g., self-determination theory) could provide a richer context for the findings.

**Response 7:**

**Thank you for this insightful suggestion. We fully agree that a single theoretical lens is insufficient to capture the complex mechanisms through which physical activity influences emotional regulation. In response, we have incorporated Self-Determination Theory (SDT) as a complementary framework to Social Cognitive Theory (SCT); please see Lines 200-215 of the revised manuscript for details.**

**Comment 8:**

Cultural Context: Provide more context on the cultural factors in Chinese universities that might influence the relationship between physical exercise and mental health.

**Response 8:**

**Thank you for prompting us to elaborate on the cultural context shaping the exercise–mental-health link among Chinese undergraduates. We have now incorporated this background in the revised manuscript (lines 178–187). We appreciate your guidance.**

**Point-by-point Responses to Reviewer 3**

**Comment 1:**

1.The introduction could benefit from a more in-depth exploration of the background surrounding college students' emotional management ability, ideally supported by concrete data or case studies for greater impact.

**Response 1:**

**Thank you very much for your valuable comments regarding the "background on college students' emotional management ability." To provide a more in-depth and compelling context, we have added further relevant content in the revised manuscript. Please refer to line 66 to 71 of the article for details.**

**Comment 2:**

When it comes to the mechanisms linking physical exercise to emotional management ability, the literature review feels underdeveloped. A more thorough examination with clearer connections would strengthen the foundation of this research.

**Response 2:**

**Thank you very much for highlighting that the review of the mechanisms linking physical exercise to emotional management ability (EMA) was underdeveloped. To strengthen the theoretical foundation, we have now added a dedicated section that thoroughly examines these pathways and cited relevant literature. Please refer to line 88 to 97 of the revised manuscript for details.**

**Comment 3:**

The theoretical foundation's coverage of psychological resilience is somewhat superficial. A deeper investigation into its role concerning emotional management ability would add significant value.

**Response 3:**

**Thank you for your comment. To provide a more rigorous and systematic account of how psychological resilience (PR) influences emotional management ability (EMA), we have added a detailed analysis of their relationship in the revised manuscript (lines 178–187).**

**Comment 4:**

The data collection section requires expansion on aspects like the questionnaire's distribution scope and recovery rate. Details on how the sample's representativeness and data reliability were ensured are also necessary.

**Response 4:**

**Thank you very much for pointing out this issue, which is extremely important for enhancing the quality and applicability of our research. For more details, please refer to lines 254-268 of the manuscript.**

**Comment 5:**

The results presentation section's tables and charts lack the desired clarity and intuitiveness. The disorganized arrangement of correlation coefficients hinders readers' ability to access key information with ease.

**Response 5:**

**Thank you very much for highlighting that "the tables and figures in the Results section lack optimal clarity and intuitiveness, and the disordered arrangement of correlation coefficients hinders readers from quickly grasping the key information." To make the findings more immediately apparent, we have standardized and improved all tables and figures. Thank you again for bringing this issue to our attention.**

**Comment 6:**

The research conclusion section doesn't provide an in-depth analysis of the mechanisms through which physical exercise influences emotional management ability via exercise adherence and psychological resilience. The conclusions drawn appear rather general and could benefit from greater specificity.

**Response 6:**

**Thank you very much for pointing out this issue. We have now provided a more thorough analysis of the mechanisms through which physical exercise influences emotional management ability via exercise adherence and psychological resilience. Please refer to lines 616-625 of the revised manuscript for the details.**

**Point-by-point Responses to Reviewer 4**

**Comment 1:**

Please confirm whether the research data can be publicly used? If there are restrictions, please explain the reasons.

**Response 1:**

**Our data contain personal information and are available only upon reasonable request to the Ethics Review Committee of Nantong University (kjc@ntu.edu.cn). Thank you again for your valuable feedback.**

**Comment 2:**

Generally, when a proper noun is mentioned for the first time, its concept should be defined, and its abbreviation should be stated, such as physical activity (PA).

**Response 2:**

**Thank you very much for your valuable suggestion that "proprietary terms should be defined and accompanied by their abbreviations when first mentioned." To align the manuscript with academic conventions and improve readability, we have now provided the full Chinese term for every proprietary concept at its first appearance in the revised manuscript, followed by its English name and abbreviation in parentheses.**

**Comment 3:**

The relationship between emotional management ability and personality traits, cognitive styles, and social support should be clearly elaborated.

**Response 3:**

**Thank you for this helpful reminder. To render the model more complete, we have added a new subsection (lines 88–97) in the revised manuscript under "Theoretical Background" that explicitly clarifies the relationships between emotional management ability (EMA) and personality traits, cognitive styles, and social support. Thank you again for your careful attention to and guidance on our work.**

**Comment 4:**

The relationship among the four variables is not clearly expressed.

**Response 4:**

**Thank you for your question. We have integrated the relationships among the four variables under the joint lens of Self-Determination Theory and Social Cognitive Theory, resulting in a clearer and more comprehensive description. Please refer to lines 202–217 of the revised manuscript for details. Thank you again for your inquiry.**

**Comment 5:**

The tables should be expressed more clearly, such as by adding percentages (%). Descriptive statistics tables should be as clear and simple as possible, and should include m (sd), kurtosis, skewness, and p-values.

**Response 5:**

**Thank you for your helpful suggestions on table clarity. We have carefully revised Table 3 accordingly; please see lines 673–674 of the revised manuscript. We appreciate your meticulous guidance.**

**Comment 6:**

Please recheck the consistency between the table content and the descriptive content, and standardize the table format according to the journal requirements.

**Response 6:**

**Thank you for highlighting the issues of internal consistency and journal formatting. We have cross-checked every entry and standardized the presentation according to the journal’s guidelines. We sincerely appreciate your meticulous attention to our manuscript.**

**Comment 7:**

Check if there are any typographical errors in the description of the direct effect 95% CI between the independent and dependent variables in Table 7.

**Response 7:**

**Thank you very much for your close attention to our manuscript. We have re-examined Table 7, corrected the error, and updated the table immediately. Your meticulous review is greatly appreciated.**

**Comment 8:**

The role of Exercise Adherence and Psychological Resilience as chain mediators in the relationship between Physical Exercise and Emotional Management Ability should be elaborated further in the discussion section.

**Response 8:**

**Thank you for your valuable suggestion that the chain-mediation pathway "exercise adherence → psychological resilience → emotional management ability" requires further elaboration. We have accordingly expanded and revised the Discussion; please see lines 471–484 and 616–624 of the revised manuscript for details.**

**Comment 9:**

In the limitations section, it should be mentioned that future research could include further exploration of the remaining variables, such as the potential influence of exercise self-efficacy and exercise motivation, which were reflected in this study.

**Response 9:**

**Thank you for this precise suggestion regarding the limitations. We have added the following sentence to the “Limitations and Future Directions” section (lines 609–612)：“Future research may address these limitations by employing longitudinal designs or experimental approaches (e.g., randomized controlled trials), including further exploration of potential variables such as exercise self-efficacy and exercise motivation to better establish causal relationships.”**
